# Toxic Effects of Bisphenol A, Propyl Paraben, and Triclosan on *Caenorhabditis elegans*

**DOI:** 10.3390/ijerph15040684

**Published:** 2018-04-05

**Authors:** María Cecilia García-Espiñeira, Lesly Patricia Tejeda-Benítez, Jesus Olivero-Verbel

**Affiliations:** 1 Environmental and Computational Chemistry Group, School of Pharmaceutical Sciences, University of Cartagena, Zaragocilla Campus, Cartagena 130015, Colombia; mgarciae@unicartagena.edu.co; 2 Biological, Toxicological and Environmental Sciences Research Group, School of Medicine, University of Cartagena, Zaragocilla Campus, Cartagena 130015, Colombia; ltejedab@unicartagena.edu.co

**Keywords:** emerging contaminants, Bisphenol A, propyl paraben, triclosan, *Caenorhabditis elegans*

## Abstract

Bisphenol A (BPA) is a ubiquitous plasticizer which is absorbed by ingestion and dermal contact; propyl paraben (PPB) inhibits the microbiome and extends the shelf life of many personal care products, whereas triclosan (TCS) is commonly found in antiseptics, disinfectants, or additives. In this work, *Caenorhabditis elegans* was used as a biological model to assess the toxic effects of BPA, PPB, and TCS. The wild type strain, Bristol N2, was used in bioassays with the endpoints of lethality, growth, and reproduction; green fluorescent protein (GFP) transgenic strains with the *hsp-3*, *hsp-4*, *hsp-16.2*, *hsp-70*, *sod-1*, *sod-4*, *cyp-35A4*, *cyp-29A2*, and *skn-1* genes were evaluated for their mRNA expression through fluorescence measurement; and quick Oil Red O (q ORO) was utilized to stain lipid deposits. Lethality was concentration-dependent, while TCS and PPB showed more toxicity than BPA. BPA augmented worm length, while PPB reduced it. All toxicants moderately increased the width and the width–length ratio. BPA and PPB promoted reproduction, in contrast to TCS, which diminished it. All toxicants affected the mRNA expression of genes related to cellular stress, control of reactive oxygen species, and nuclear receptor activation. Lipid accumulation occurred in exposed worms. In conclusion, BPA, PPB, and TCS alter the physiology of growth, lipid accumulation, and reproduction in *C. elegans*, most likely through oxidative stress mechanisms.

## 1. Introduction

Endocrine-disrupting chemicals (EDCs) are natural or synthetic compounds and are called xenoestrogens because of their capability to disrupt endocrine functions by mimicking or blocking endogenous hormones [1,2]. For many years, EDCs have been widely introduced into the environment and to the human food chain, exposing living organisms to their actions [3,4]. Among the EDCs, bisphenol A, propyl paraben, and triclosan are some of the most important chemicals due to their extended use.

Bisphenol A (2,2-bis (4-hydroxyphenyl) propane) (BPA) is widely used in the production of polycarbonate plastics, epoxy resins, thermal paper, paints, water-pipes, electronic equipment, toys, packaging, bottles, medical devices, surface coatings, printing inks, flame retardants, laptops, mobile phones, electronic devices, dental sealants, and laboratory and hospital equipment, among others [5,6,7,8,9]. Some of these applications were the causes of human exposure to BPA through food and drinks [10,11], or by inhalation and dermal contact [12,13,14]. Furthermore, due to its wide range of industrial uses, BPA is released into the environment, raising concerns regarding aquatic and terrestrial ecosystems as BPA is now present in surface water, atmospheric dust, sediment, and biota [14,15,16]. BPA has been identified in house dust at concentrations between 0.2 and 17.6 μg/g, in air samples at an average concentration of 0.51 ng/m^3^, and in air samples from workplace plastics (208 ng/m^3^) [17] at concentrations of 2.4–3.59 ng/m³ [18]. In human hair, the mean BPA concentration amounts to 411.2 ng/g [19].

BPA binds to various receptors such as estrogen and androgen, aryl hydrocarbon receptor (AhR), and peroxisome proliferator-activated receptor (PPAR), all of which are associated with the endocrine system [20]. Moreover, BPA is able to disrupt the function of sex hormones, leptin, insulin, and thyroxin. It is also known that, even at a nanomolar level, BPA is able to induce genotoxic, mutagenic, hepatotoxic, immunotoxic, neurotoxic, teratogenic, and carcinogenic effects [21,22]. More recently, it has been suggested that BPA increases the risk of obesity, diabetes, and heart disease in humans and is related to epigenetic modifications [9,23,24,25,26].

Parabens are alkyl esters of *p*-hydroxybenzoic acid, an excellent preservative with antimicrobial activity, and they are used to control molds and yeasts in food, beverages, and cosmetic and pharmaceutical products due to their relatively low toxicity and safety [27,28]. Human exposure to parabens may take place through ingestion, inhalation, or dermal absorption, and several parabens have been suggested to interfere with endocrine signaling and to stimulate adipocyte differentiation [29]. Propyl *p*-hydroxybenzoate, commonly referred to as propyl paraben (PPB), is a member of this family which most inhibits microbial growth and extends the shelf life of a range of consumer products [30]. PPB is a stable molecule over the pH range, and it is soluble enough in water to produce an effective concentration in an aqueous phase [31]. It is readily biodegradable under aerobic conditions, and its potential for bioaccumulation is low to moderate due its low octanol–water partition coefficient [32,33]. It is known that the estrogenic effect of PPB is approximately 10,000-fold lower than 17β-estradiol, but is equal in potency to 4-nonylphenol [34]. Despite the fact that parabens are considered relatively safe compounds with a low bioaccumulation potential [35], their detection in human fluids [36,37,38,39,40,41] and human tumors [42,43] have demonstrated in several in vivo and in vitro screening tests that parabens have endocrine-disrupting activity that may represent a potential risk to human health [44,45,46]. PPB was detected in human urine at concentrations of 75.3 μg/L and in cord blood plasma samples at concentrations below 0.27 μg/L [47]. PPB may alter the viability of human sperm, and in animals, some studies showed that its exposure stimulates cell proliferation in the forestomach of rats [48,49]. Meanwhile, a study involving hamsters reported that PPB augmented the labeling index of their urinary bladder epithelium [50]. In addition, other investigations have concluded that exposure of rats and mice to PPB negatively affected the secretion of testosterone [51] and caused significant vitellogenin induction in male rainbow trout, [52] in medaka [53], and in *Danio Rerio* [54]. Consequently, the unwitting and continued exposure of this preservative exerts deleterious effects on humans and environmental health.

Triclosan (TCS) is widely used in personal care products such as antiseptics and disinfectants, or in additives used in clinical applications, cosmetics, household cleaners, plastics, paints, and textiles, among others. TCS has been detected in environmental samples such as waters, sediments, and soils. Due to its extensive use, persistence, low water solubility, and high octanol–water partition coefficient, TCS can accumulate in soils and sediments, and it has been found in wastewaters, surface waters, sediments and biological samples [55], and wastewater treatment effluents [56,57]. For instance, in fish muscle, TCS concentrations have been registered at <0.2–3.4 ng/g [58]. Underground water has been reported to have up to 0.10 nM TCS [59], whereas untreated surface waters had reported concentrations between 7.9 and 39 nM [60]. At high concentrations, TCS is a biocide with several cytoplasmic and membrane targets, whereas at minor concentrations found in commercial products, it inhibits bacterial fatty acid synthesis [61].

Some studies have reported the toxicity of TCS in different organisms, including *Daphnia magna* [62,63,64] *Thamnocephalus platyurus* [65], *Ampelisca abdita*, *Americamysis bahia* [66], *Artemia salina* [67], *Eisenia fetida* [30,68,69], *Achatina fulica* [70] *Dreissena polymorpha* [71], *Chironomus riparius* [72,73,74], *Chlorococcum* sp. [75], *Chlamydomonas reinhardtii* [74], *Gammarus pulex* [76], *Tigriopus japonicas* [77], *Bufo gargarizans* [78], and *Chlamydomonas reinhardtii* [79], among others. These studies have been related to several toxicity endpoints and mechanisms such as oxidative stress and changes in gene expression. Some authors studied the effects of TCS as an endocrine disruptor; for instance, studies on *Chironomus riparius* showed that TCS may alter the transcriptional activity of endocrine-related genes [74]. In bioassays using *Daphnia magna*, TCS induced a significant decrease in the number of neonates [64]. Moreover, TCS reduced the fecundity of *Tigriopus japonicas* [77] and has been associated with alterations in the endocrine function in humans [80]. More recently, it has been reported that TCS causes an acute toxicity in *C. elegans* and also an induced reduction in its reproduction, lifespan, and delay in its hatching [81].

In this work, the nematode *Caenorhabditis elegans* was used as a biological model to assess the toxic effects of BPA, PPB, and TCS. This organism is widely used as a model to assess the toxicity of several compounds such as metals [82,83], pesticides [84], nanoparticles [85,86], and emerging pollutants [87]. *C. elegans* has been a popular biological model that has been used to investigate the effects of toxicants through several endpoints such as body length, development, and brood size, among others [88], and it is an excellent model to evaluate reproductive toxicity [89]. Lethality was used to establish the sublethal concentrations of these compounds. Growth, reproduction, changes in gene expression, and lipid accumulation were endpoints selected to analyze endocrine disruption.

## 2. Materials and Methods

### 2.1. Nematodes and Bacteria

The *C. elegans* wild-type strain Bristol N2 was used in the bioassays of growth and fertility. Green fluorescent protein (GFP) transgenic nematodes integrated to genes coding for heat shock proteins (*hsp-3*, *hsp-4*, *hsp-16.2*, and *hsp-70*), antioxidant enzymes (*sod-1* and *sod-4*), biotransformation enzymes (*cyp-35A4* and *cyp-29A2*), and transcription factor (*skn-1*), were used to determine changes in gene expression. *Escherichia coli* OP50 was used as nourishment in K agar that was prepared with KCl, NaCl, agar, peptone, cholesterol, CaCl_2_, and MgSO_4_. An age-synchronized population of worms was obtained by bleaching them in an alkaline solution [39].

### 2.2. Solutions and Exposure

**Figure 1:**
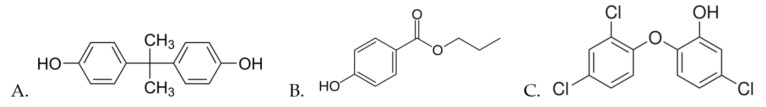
Structural formula of pollutants. (**A**) Bisphenol A; (**B**) Propyl paraben; (**C**) Triclosan.

The reagents that were purchased from Sigma Aldrich were BPA (CAS Number 80-05-7; Sigma, St. Louis, MO, USA), PPB (CAS Number 94-13-3; St. Louis, MO, USA), and TCS (CAS Number 3380-34-5; Sigma, St. Louis, MO, USA). The structural formula of each compound is displayed in Figure 1. Age-synchronized nematodes were exposed to 0.05–5000 μM of each compound. A K medium (NaCl 52 mM and KCl 32 mM in ultra-filtered water) [39] was utilized as a solvent and as a control. Four replicates were performed per sample, and each experiment was repeated three times.

### 2.3. Lethality Assay

Nematodes in the L4-larval stage were exposed to toxic solutions. Approximately 10 ± 1 worms were used for treatment. After 24 h, the number of live and dead worms was counted through visual inspection using a dissecting microscope. Worms were scored as dead when physical stimuli failed to generate any response [39,90].

### 2.4. Growth Assay

The growth of nematodes was assessed in larval age L1 after 48 h exposure to toxic solutions. *E. coli* OP50 was inoculated as a source of food. After their exposure to toxic solutions, the nematodes were heated to 50 °C, and this aimed to make their bodies adopt a straight line. Body length and width were then measured by analyzing a photograph recorded by a dissecting microscope and using the software Image J. The width and body length ratio (WBR) was estimated as the quotient between the body length and the width. About 30 nematodes were examined per treatment [91,92,93,94].

### 2.5. Reproduction Assay

Nematodes previously exposed for 24 h to toxic solutions were set individually in K agar plates seeded with *E. coli* OP50, and the number of offspring at all stages was counted after 24 h. About 10 nematodes were examined per treatment [95,96,97,98].

### 2.6. Gene Expression through Fluorescence Measuring

The effects on gene expression were monitored utilizing GFP transgenic *C. elegans* strains containing the *hsp-3*, *hsp-70*, *sod-1*, *sod-4*, *gpx-4*, and *gpx-6* genes. Equal aliquots of nematodes on all larval stages were placed into black, non-fluorescent, U-bottomed, 96-well microplates with the toxic solutions of BPA, TCS, and PPB. The plates were incubated at 15 °C, and after 24 h, the fluorescence intensity was quantified using a plate reader (Fluoroskan Ascent, Thermo Scientific, Waltham, MA, USA) with excitation/emission filters of 485/525 nm [99,100,101,102,103]. The relative fluorescence was calculated as the quotient between the solution’s fluorescence and that of the control [90].

### 2.7. q-ORO Assay

The q-ORO assay was done as described [104]. Age-synchronized Bristol N2 wild type nematodes in the L4-larval stage were exposed for 24 h to toxic solutions of BPA, PPB, and TCS. To prepare the dye solution, a 0.5% oil red O (MP, Cat. No. 155984) stock solution was prepared in high-quality 100% isopropanol, incubated at room temperature for a day, and then filtered through a 0.45 µm filter. The stock was freshly diluted to 60% with filtered water the day before its use, and it was then incubated at room temperature overnight. The stock was filtered through a 0.45 µm filter. Two hundred microliters of high-quality 60% isopropanol was added to the worms in the 96-well PCR plates (Thermo Scientific, Waltham, MA, USA). Worms were settled to the bottom of the wells, and then they were aspirated with up to 175 µL of buffer. Two hundred microliters of freshly filtered ORO working solution was added, which sealed the plates. Worms were stained for 6–18 h at 25 °C; after 6–18 h, the supernatant was aspirated. Then, 100 µL of 0.01% Triton X-100 was added in the S buffer. Images were recorded by using an optic microscope [104].

### 2.8. Statistical Analysis

Data are presented as mean ± standard error. Normality and variance homogeneity were verified using the Kolmogorov–Smirnov and the Bartlett tests, respectively. Significant differences between means were determined with one-way analysis of variance (ANOVA) test. The Dunnett test was applied to compare each solution with the control. The significance level or criterion of significance was set at *p* < 0.05. Statistical analyses were performed with SPSS for Windows (Version 23, Statistical Package for the Social Sciences, Inc., Chicago, IL, USA) and Graphpad Prism (Version 5.0, GraphPadSoftware, San Diego, CA, USA).

## 3. Results

### 3.1. Lethality

**Figure 2:**
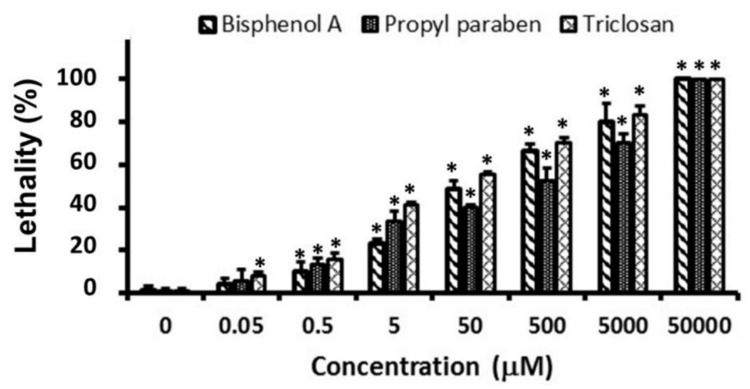
Lethality of *C. elegans* exposed to bisphenol A, propyl paraben, and triclosan. * Significant difference compared to control (*p* < 0.05).

The results of the lethality bioassay are displayed in Figure 2. The lethality was concentration-dependent, and the LC50 after 24 h exposure for BPA, PPB, and TCS were 113.5, 261.7, and 43.2 µM, respectively (Table S1). At concentrations greater than 0.5 µM, all compounds caused lethality, with statistical differences being related to the control. At lower concentrations, only TCS (0.05 µM) was bioactive.

### 3.2. Growth

**Figure 3:**
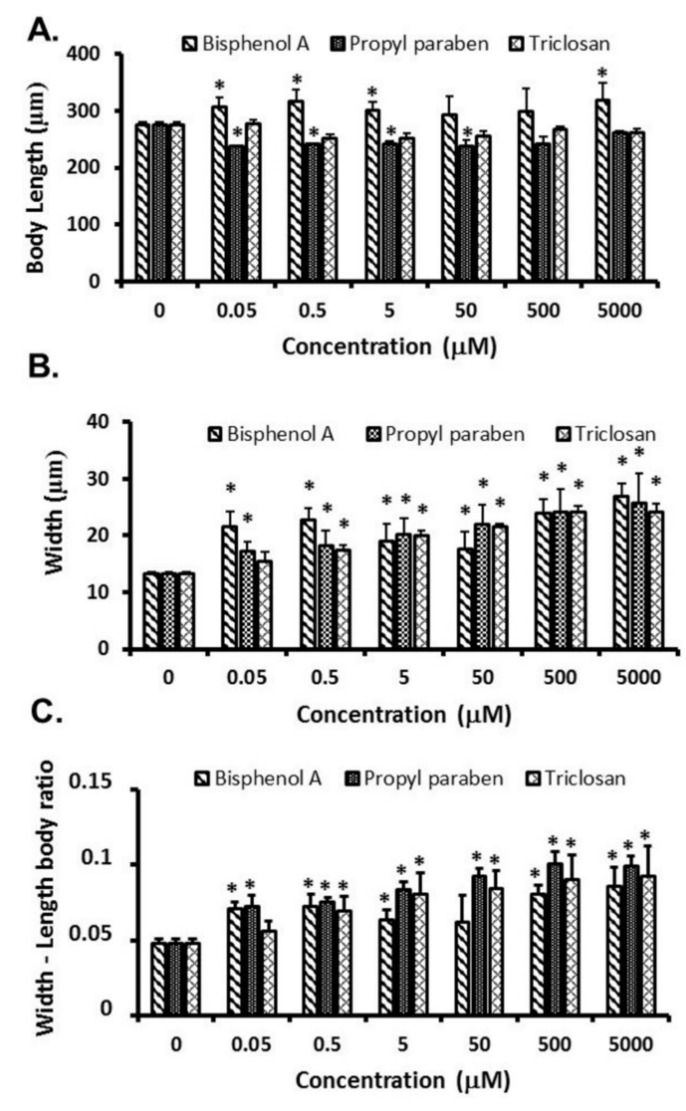
Changes in body length (**A**), width (**B**), and width/length ratio (**C**) in *C. elegans* exposed to bisphenol A, propyl paraben, and triclosan. * Significant difference compared to control (*p* < 0.05).

Changes in the body length, the body width, and the body width–length ratio of nematodes exposed to BPA, PPB, and TCS solutions are shown in Figure 3. Body length was slightly increased by BPA but was not concentration-dependent. In contrast, PPB reduced the body length, while TCS did not have an effect on this parameter. All the toxic chemicals moderately increased the body width, without a clear relationship with concentration, although the response elicited by BPA was bimodal. The relation between the body width and the body length of the nematodes was moderately increased by an exposure to the tested chemicals, but PPB was the most active, suggesting a probable association with obesity in the *C. elegans* model.

### 3.3. Reproduction

**Figure 4:**
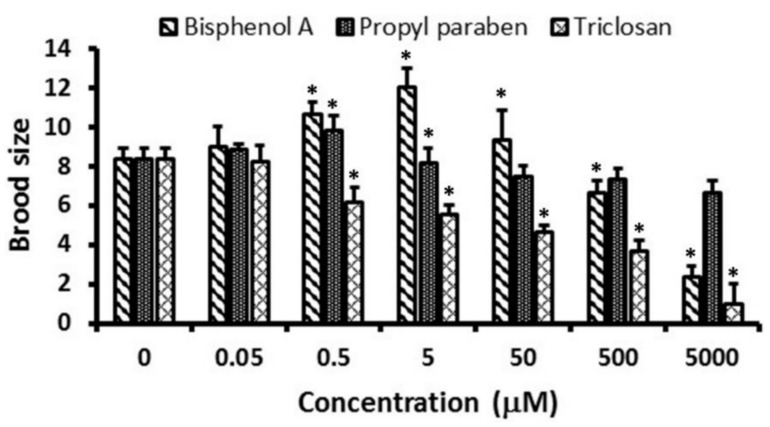
Brood size of *C. elegans* exposed to bisphenol A, propyl paraben, and triclosan solutions. * Significant difference compared to control (*p* < 0.05).

The brood size of nematodes exposed to BPA, PPB, and TCS solutions are shown in Figure 4. The greatest brood size after BPA exposure was reached at 5 µM; afterwards, it decreased in response to higher concentrations. Similarly, PPB increased the brood size until 0.5 µM, with declining effects at greater concentrations. In contrast, TCS decreased the brood size following a concentration-dependent trend.

### 3.4. Changes in Gene Expression

**Figure 5:**
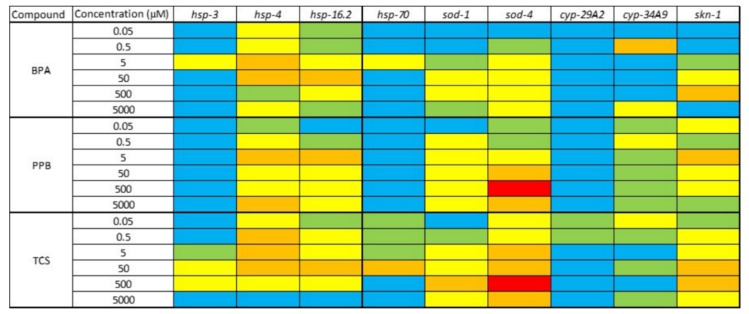
Changes in the mRNA expression profiles of evaluated genes measured in relation to control. Blue: <2-fold; Green: 2–3-fold; Yellow: 3–4-fold; Orange: 4–5-fold; Red: >5-fold.

The relative changes in gene expression in *C. elegans* carrying *gfp*-reporter genes are displayed in Figure 5, and graphs are presented in Figures S1–S9 in Supplementary Material. The most sensitive genes, in descending order, were *sod-4*, *hsp-4*, *hsp-16.2*, and *skn-1*. These genes increased their expression after their exposure to all the compounds, indicating a toxic response related to the generation of reactive oxygen species (ROS). There was no evidence of concentration dependence in these results. Furthermore, low concentrations caused the overexpression of some genes; for instance, BPA at concentrations of 0.05 and 0.5 µM caused a 3-fold expression of *hsp-4*. However, high concentrations also affected the expression of several genes such as *sod-4*, which showed a 5-fold upregulation after its exposure to PPB and TCS at concentrations of 500 µM when compared to the control.

### 3.5. q-ORO Stain

**Figure 6:**
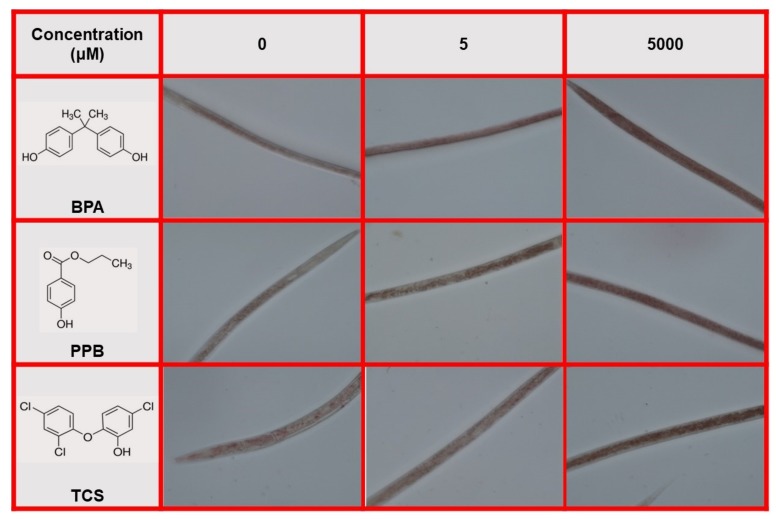
q-ORO staining of nematodes exposed to BPA, PPB, and TCS.

Representative images of nematodes that were exposed to the BPA, PPB, and TCS solutions and stained with q-ORO are displayed in Figure 6. All the tested chemicals caused lipid deposition inside the bodies of exposed nematodes. According to the intensity of the color recorded in the images, the deposits formed show an increasing trend related to concentration. BPA caused more lipid accumulation, followed by PPB and TCS. This result is consistent with the changes in the body width–length ratio that were registered in the worms after their exposure to these molecules.

## 4. Discussion

Exposure to BPA, PPB, and TCS alters the physiology of *C. elegans* in terms of growth, reproduction, and gene expression. In this work, TCS exerted the greatest acute toxicity on *C. elegans*, followed by BPA and PPB. There is extensive information on BPA toxicity for several organisms. For example, in the snail, *Pomacea lineata*, the 96 h LC50 was 11.1 mg/L [105], whereas in the sea squirt, *Ciona intestinalis*, the LC50 was 5.4 µM [106]. Other authors have also reported toxicity data for BPA on *C. elegans*, with a 24 h LC50 value of 1422 µM (324.7 mg/L) [107]. However, they used 1-day-old (L1/L2) larvae, which may explain the observed difference in the order of magnitude in toxicity. Propyl paraben was less toxic than BPA, although organisms such as *D. magna* and *Pimephales promelas* were more sensitive to PPB exposure than *C. elegans*, with LC50 values of 12.3 mg/L and 9.7 mg/L, respectively [108]. Finally, the 24 h LC50 for TCS was 43.2 µM (9.84 mg/L), comparable to 3.65 mg/L as previously reported [81]. Other model organisms, such as *Artemia salina*, *Thamnocephalus platyurus*, and *Chironomus riparius* have showed LC50 values after 24 h exposure of 171, 470, and 3428 μg/L TCS, respectively [65,67,74].

The effect of BPA on the growth of *C. elegans* varied according to the level of exposure, and as presented here, the concentration–response relationship is not always monotonic [109,110]. The effects of PPB and TCS have not been reported yet on the growth of *C. elegans*. However, other organisms have been tested; for example, TCS at a level below 0.8 µM did not have significant effects on the body length of *D. magna* [62]. In regards to PPB, the lowest concentration associated with the growth of *C. elegans* that has had an observed effect has been estimated to be 0.4 mg/L (2.2 µM) for *D. magna* and 2.5 mg/L (13 µM) for *P. promeras* [108].

The reproductive outcome of *C. elegans* after its exposure to tested chemicals suggests that endocrine disruption had occurred. For instance, the effect of BPA on nematode reproduction is exemplified by a *non*-*monotonic*, inverted U-shape curve, where the effects of increasing concentrations of the compound appear to increase up to a peak and then decrease [111]. This nonlinear concentration–response relationship has also been described for some endpoints of BPA studies on *C. elegans* [112,113,114]. It has also been proven that the exposure to BPA decreases fecundity [109]. This effect and others associated with reproduction have been related to strong negative effects of this plasticizer on the germline function of *C. elegans* [114]. Although PPB induced a small increase in broad size at 0.5 µM, it seems to follow a slight concentration-dependent reduction in the reproduction of *C. elegans.* Interestingly, the effect of TCS was definitively inhibitory and dependent on concentration, as previously reported for this model [81] and also in *D. magna* [62].

Some studies about the changes in gene expression of *C. elegans* that were exposed to BPA have reported that the expression of *hsp-70* exhibited a hormetic decrease, while *hsp-16.2* showed a dose-dependent increase, which was also observed for *sod-3* expression [109]. The mechanisms of action by which xenobiotics regulate Superoxide dismutase (SOD) enzymes in *C. elegans* are not completely elucidated. The activation of the antioxidant system in *C. elegans*, which includes SOD, among others, can be rather complex, and the deletion of free radicals and their toxic effects may occur through a multistep process [115]. *C. elegans* is highly susceptible to oxidative stress, and even its manipulation could generate stress and influence the internal redox balance [116]. The fact that all tested xenobiotics overexpress genes coding for molecules involved in the biological defense processes, such as *sod-1* and *sod-4*, may result from the organism avoiding ROS formation. However, once ROS levels pass the required threshold, the defense against oxidative stress decreases, leading to toxicity. The activation of *hsp-70* transcription suggests protection against protein oxidation and neuronal damage [117], which suggests that these chemicals play a role in oxidative damage.

Bisphenol A, PPB, and TCS caused the overexpression of *cyp-34A9*. This gene encodes monooxygenases, one of the cytochrome P450 proteins which catalyze reactions involved in drug metabolism and in the synthesis of steroid hormone signaling [118]. Moreover, the CYP system is one of the main targets of different nuclear xenobiotic receptors, directing different pathways of xenobiotics metabolism [119,120]. These processes may also have an indirect link to endocrine disruption by binding to estrogenic hormone receptors in the worm [121], especially since BPA is an estrogen-receptor ligand [122] that has been shown to transcriptionally activate the CYP2C9 promoter [123].

Bisphenol A, PPB, and TCS also up-regulated *skn-1*. This gene encodes the SKN-1 proteins, which are required for longevity and oxidative stress resistance in *C. elegans* [124]. There are indeed some similarities between the pattern expression of *sod-1*/*sod-4* and *skn-1* genes after their exposure to the tested compounds (Figure 5), which corresponds with the role of this gene in oxidative stress responses [125].

One of the most interesting findings in this work was that BPA, PPB, and TCS promoted lipid accumulation in *C. elegans*, a process verified through the fixation of q-ORO to lipidic deposits. An important aspect is that there are no reports of obesogenic effects, in terms of lipid accumulation, on *C. elegans*, as a marker of endocrine disruption elicited by these compounds. However, BPA has been recognized as an obesogen, promoting the adipogenesis, the lipid dysregulation, and the inflammation of adipose tissue [126]. It should be emphasized that more studies are needed to verify these results and the mechanisms involved in the lipid accumulation process carried out by the nematode.

## 5. Conclusions

Triclosan generated more acute toxicity than bisphenol A and propyl paraben in *C. elegans*. Bisphenol A and propyl paraben increased the brood size of *C. elegans*, and triclosan had a negative effect on reproduction. All compounds increased the expression of stress response genes such as *sod-4* and *skn-1*, which are related to the oxidative stress response; *hsp-4* and *hsp-16.2*, which are associated with cellular stress; and other genes such as *cyp-34A9*, which may be a response to their interaction with nuclear xenobiotic receptors. These molecules also increased the lipid accumulation in *C. elegans.* Taken together, these results *suggest* that these chemicals promote endocrine disruption mechanisms at levels which influence reproduction and obesity.

## Supplementary Materials

The following are available online at http://www.mdpi.com/1660-4601/15/4/684/s1. Table S1. LC50 values and confidence limits for tested compounds after 24 h exposure. Figure S1. Effects of BPA, PPB and TCS on *hsp-3* gene expression measured as GFP fluorescence. Figure S2. Effects of BPA, PPB and TCS on *hsp-4* gene expression measured as GFP fluorescence. Figure S3. Effects of BPA, PPB and TCS on *hsp-16.2* gene expression measured as GFP fluorescence. Figure S4. Effects of BPA, PPB and TCS on *hsp-70* gene expression measured as GFP fluorescence. Figure S5. Effects of BPA, PPB and TCS on *sod-1* gene expression measured as GFP fluorescence. Figure S6. Effects of BPA, PPB and TCS on *sod-4* gene expression measured as GFP fluorescence. Figure S7. Effects of BPA, PPB and TCS on *cyp-29A2* gene expression measured as GFP fluorescence. Figure S8. Effects of BPA, PPB and TCS on *cyp-34A9* gene expression measured as GFP fluorescence. Figure S9. Effects of BPA, PPB and TCS on *skn-1* gene expression measured as GFP fluorescence.
