# Supplementary material for: Toxic Effects of Bisphenol A, Propyl Paraben, and Triclosan on Caenorhabditis elegans
Source: Int J Environ Res Public Health. 2018 Apr 5;15(4):684. doi: 10.3390/ijerph15040684 (PMC5923726; doi:10.3390/ijerph15040684)
Supplement: Supplementary file 1 [file ijerph-15-00684-s001.pdf]

Supplementary Material

Table S1. LC50 values and confidence limits for tested compounds after 24 h exposure.

Compound	24 h-LC50 (µM)	Lower limit (µM)	Upper limit (µM)
**BPA**	113.5	25.3	508.9
**PPB**	261.7	42.0	1631.1
**TCS**	43.2	8.6	217.1

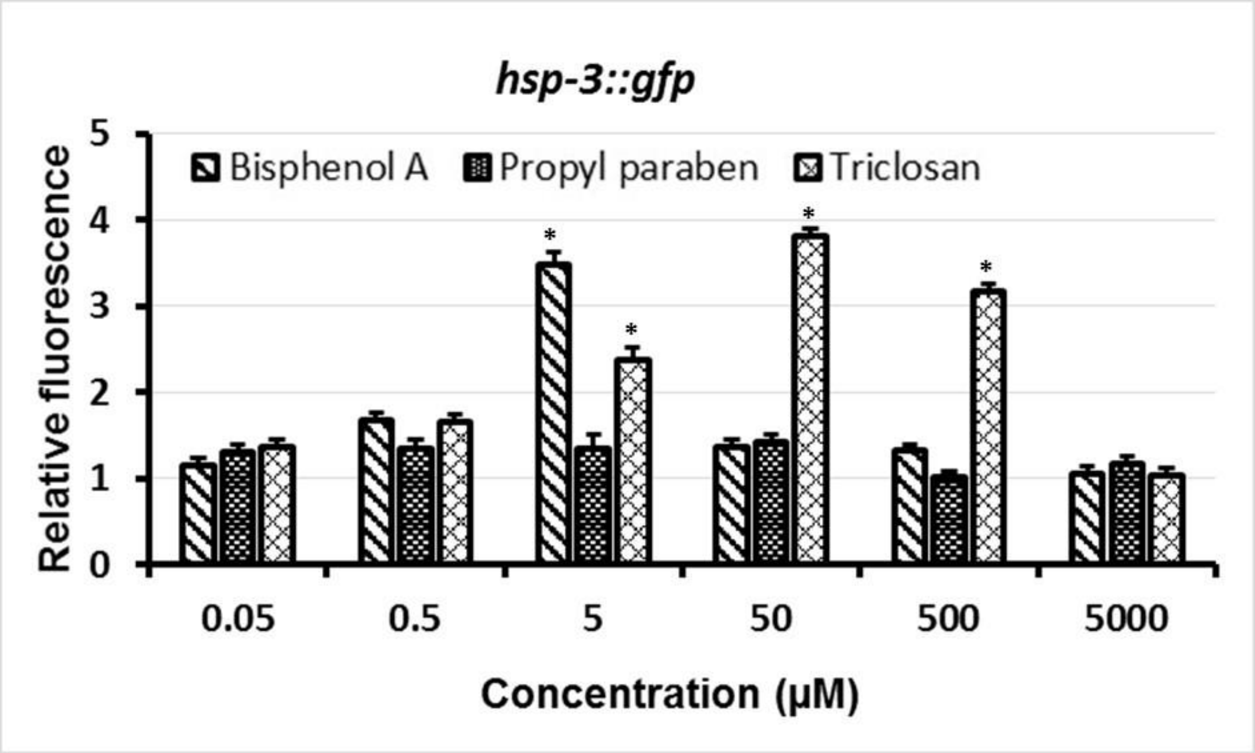

Figure S1. Effects of BPA, PPB and TCS on *hsp-3* gene expression measured as GFP fluorescence. *-Significant difference with control ($p < 0.05$).

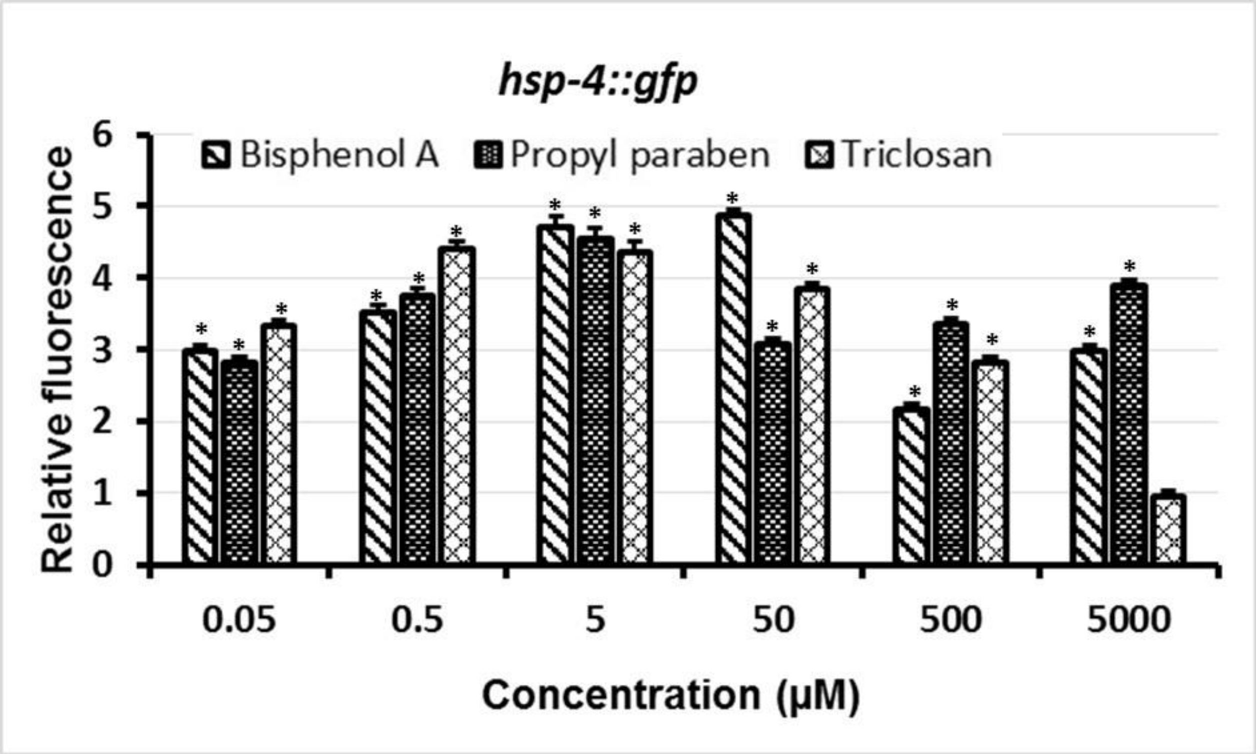

Figure S2. Effects of BPA, PPB and TCS on *hsp-4* gene expression measured as GFP fluorescence.

*. Significant difference with control ($p < 0.05$).

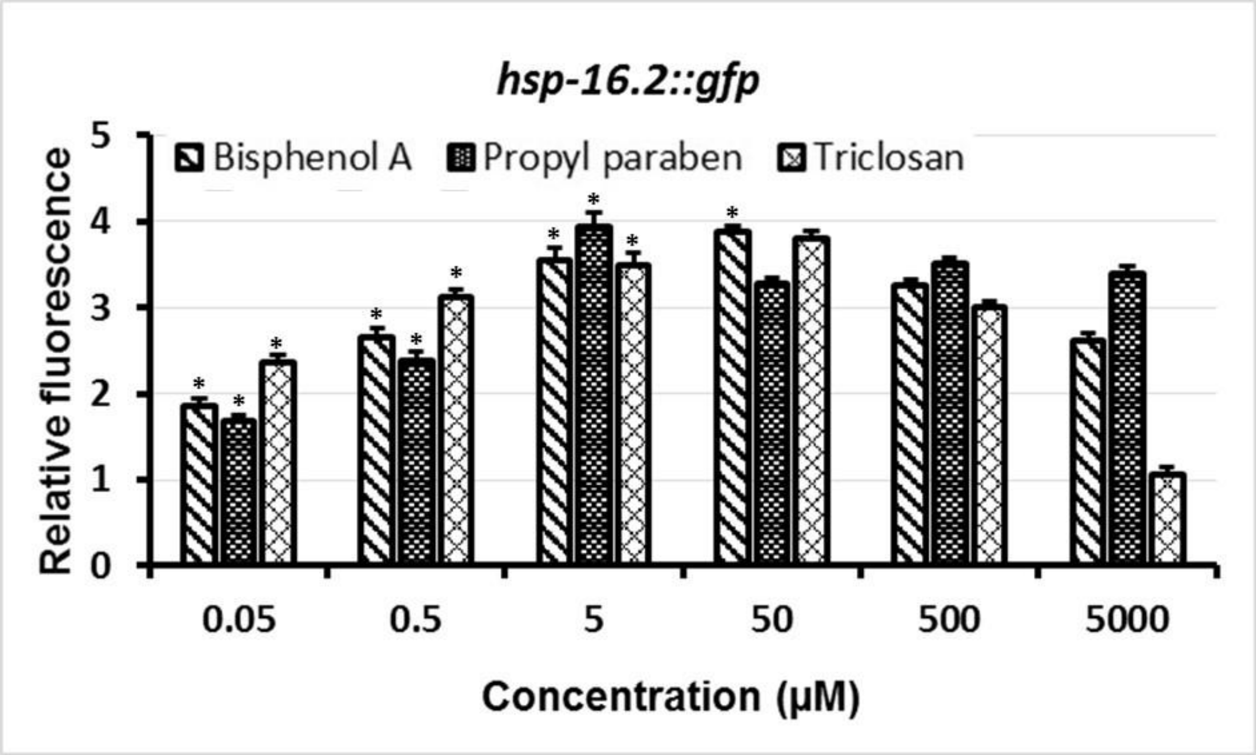

Figure S3. Effects of BPA, PPB and TCS on *hsp-16.2* gene expression measured as GFP fluorescence.

*. Significant difference with control ($p < 0.05$).

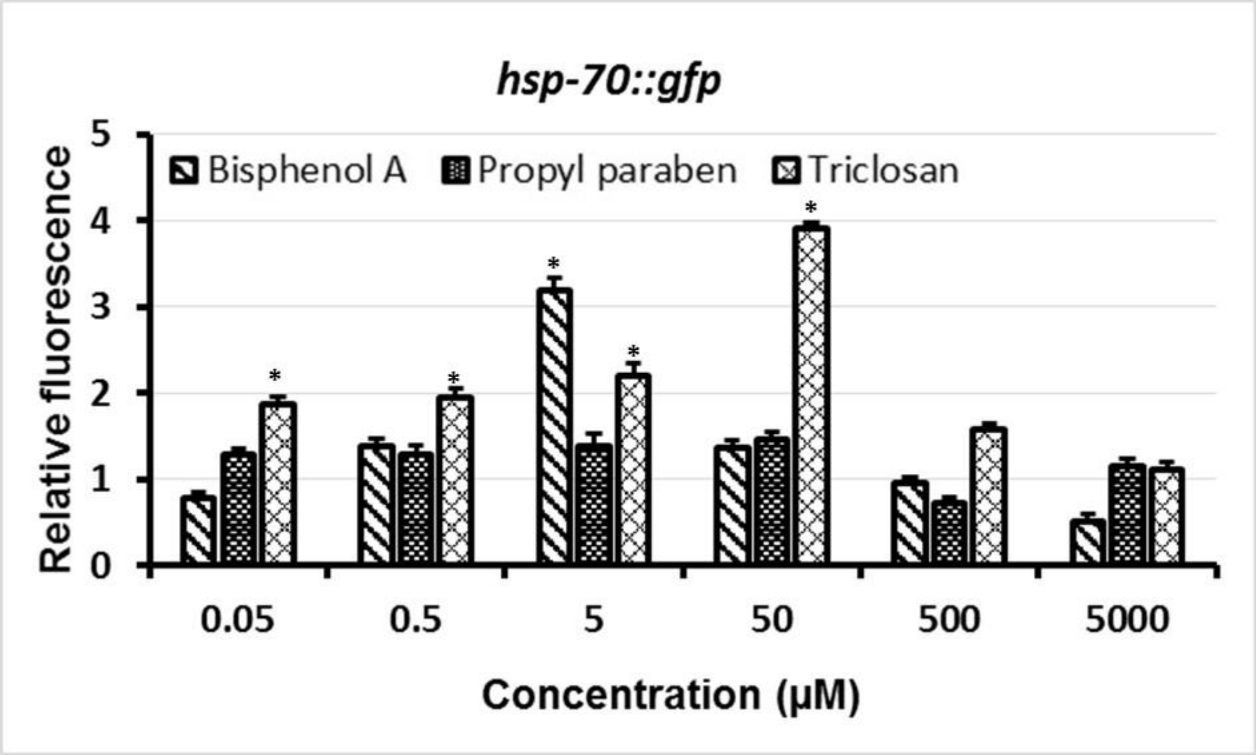

Figure S4. Effects of BPA, PPB and TCS on *hsp-70* gene expression measured as GFP fluorescence.

*. Significant difference with control ($p < 0.05$).

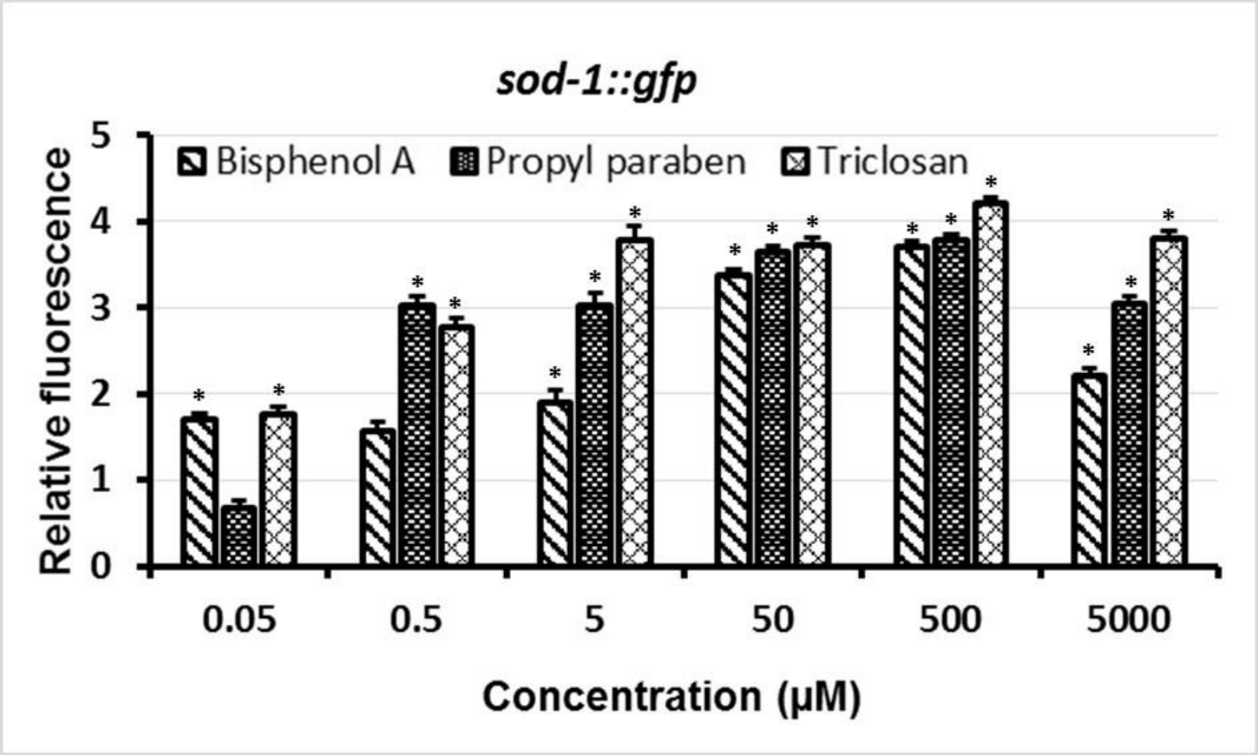

Figure S5. Effects of BPA, PPB and TCS on *sod-1* gene expression measured as GFP fluorescence.

*. Significant difference with control ($p < 0.05$).

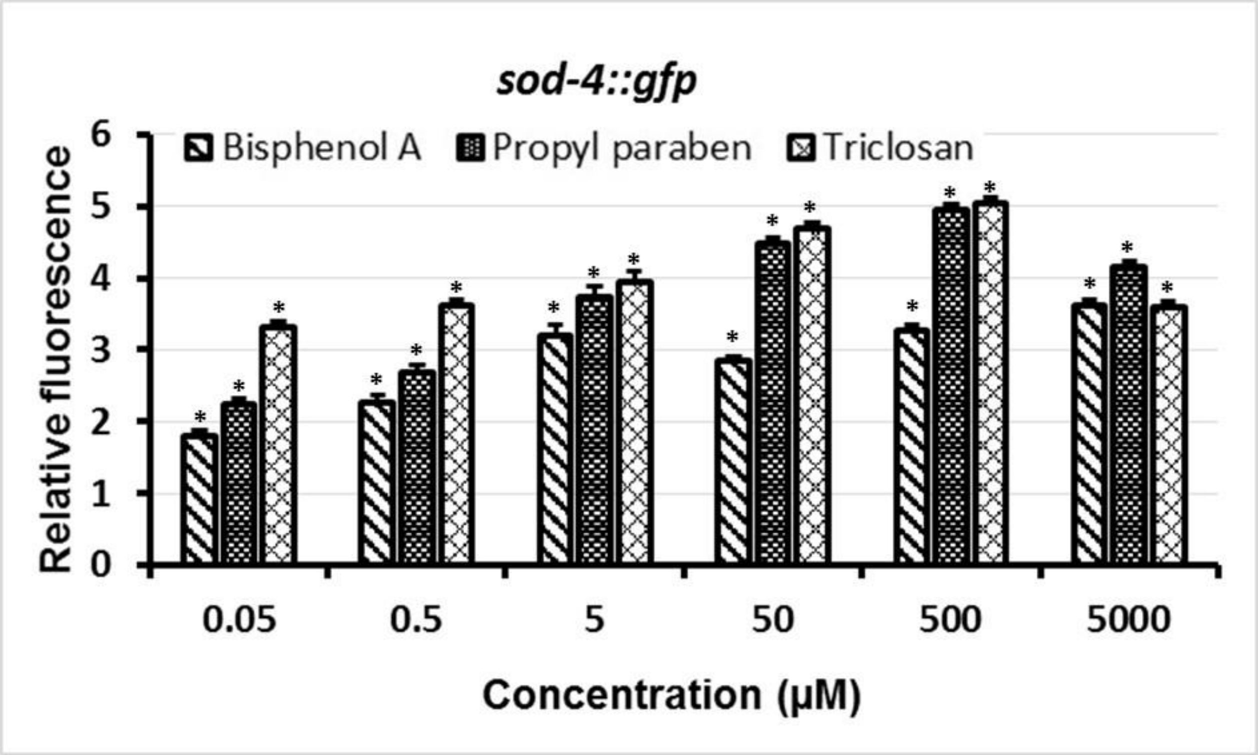

Figure S6. Effects of BPA, PPB and TCS on *sod-4* gene expression measured as GFP fluorescence.

*. Significant difference with control ($p < 0.05$).

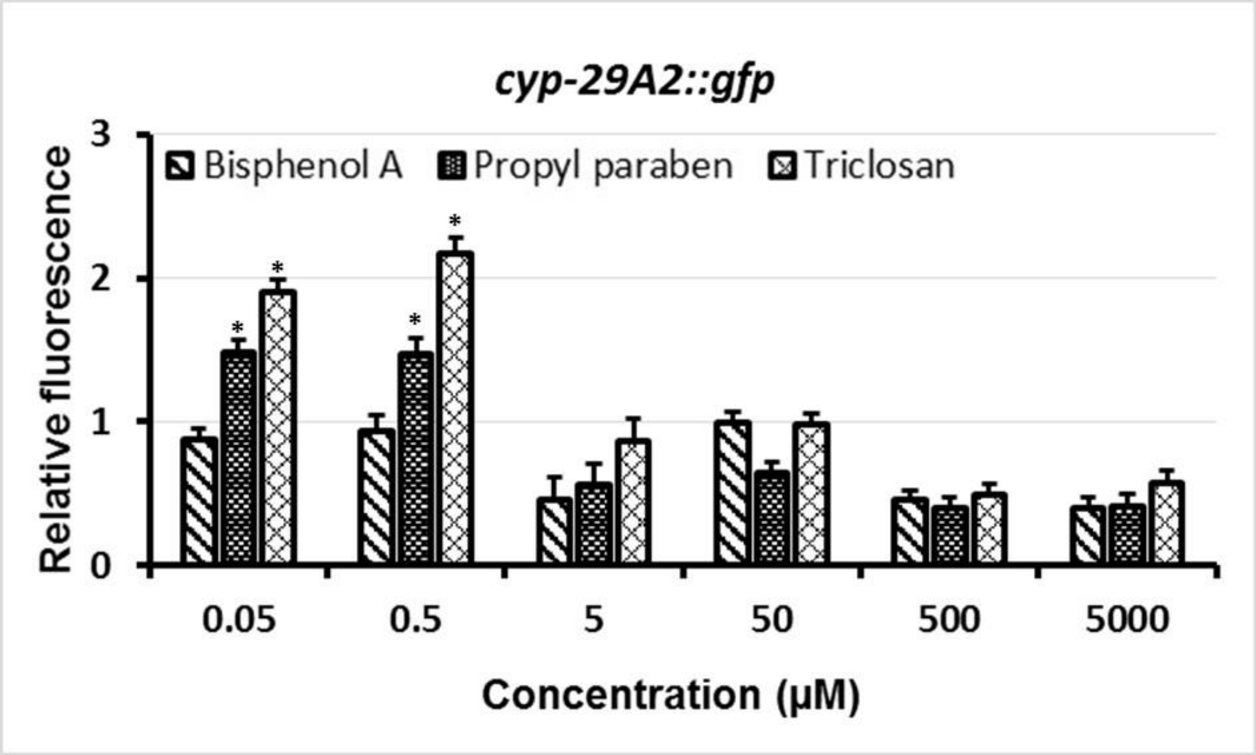

Figure S7. Effects of BPA, PPB and TCS on *cyp-29A2* gene expression measured as GFP fluorescence.

*. Significant difference with control ($p < 0.05$).

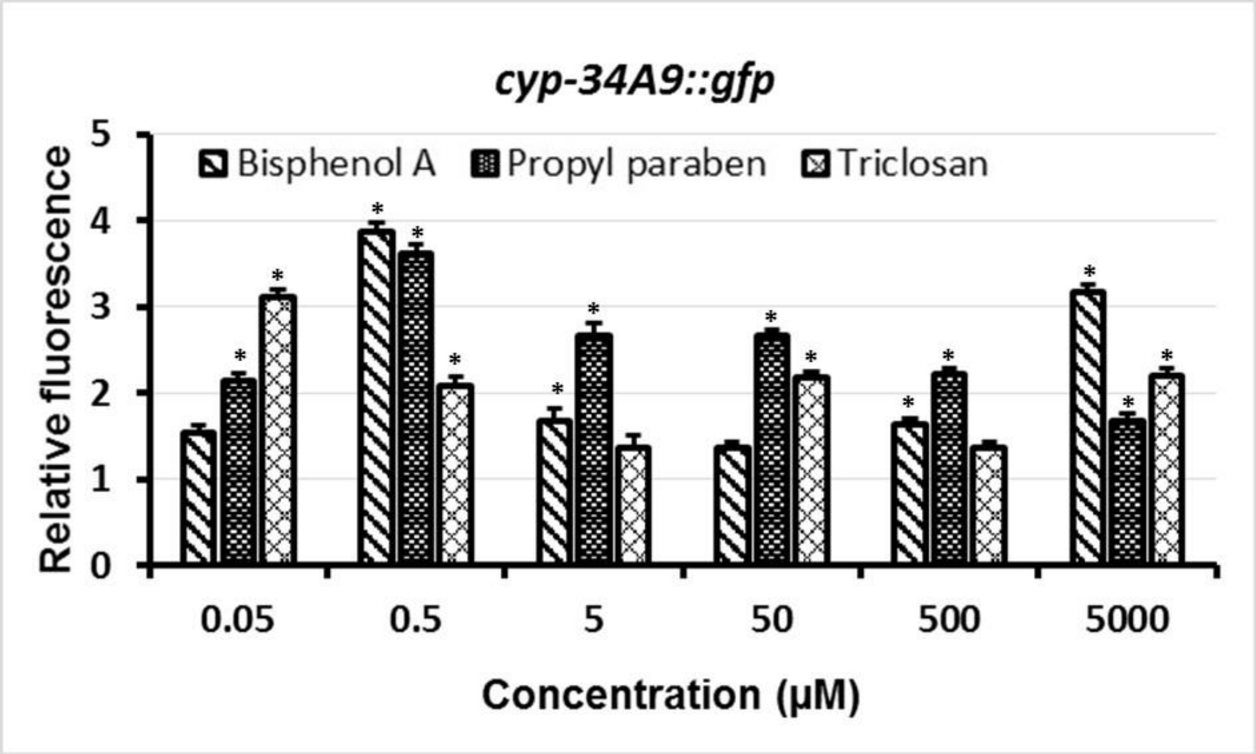

Figure S8. Effects of BPA, PPB and TCS on *cyp-34A9* gene expression measured as GFP fluorescence.

*. Significant difference with control ($p < 0.05$).

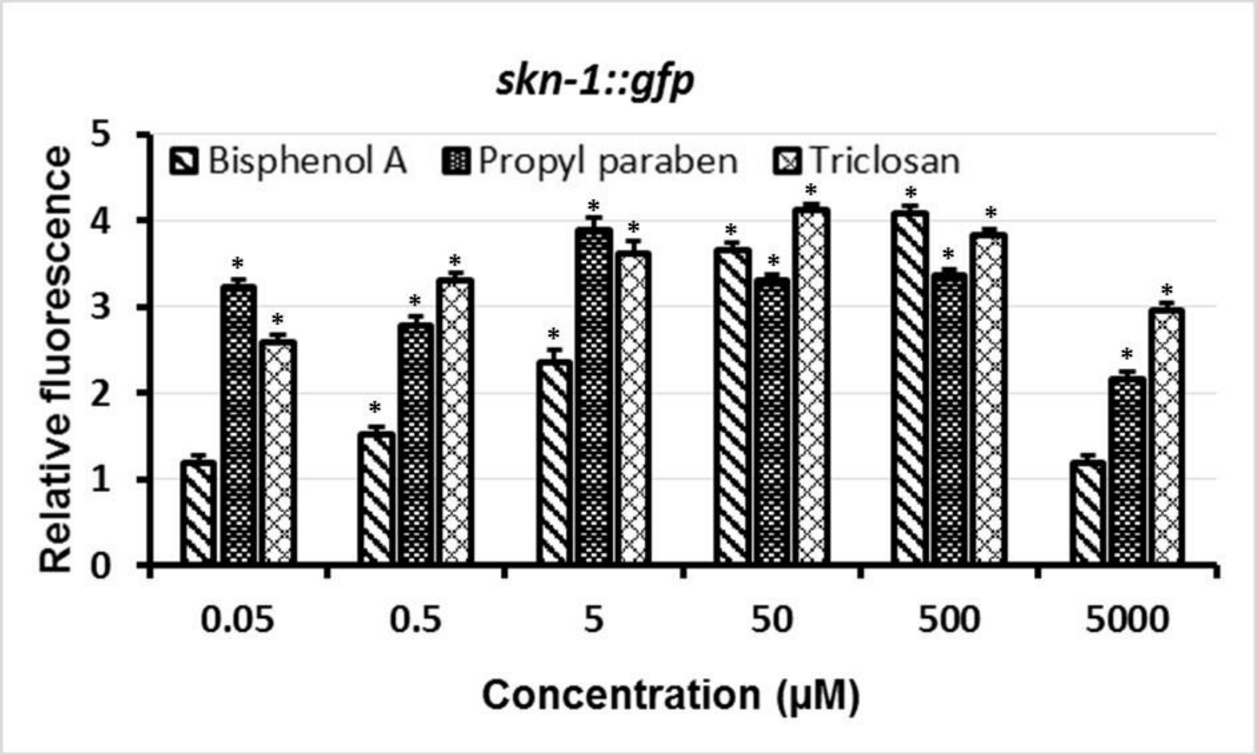

Figure S9. Effects of BPA, PPB and TCS on *skn-1* gene expression measured as GFP fluorescence.

*. Significant difference with control ($p < 0.05$).
